# Conference Reports: NORTH AMERICAN INTEGRATED SERVICES DIGITAL NETWORK (ISDN) USERS’ FORUM (NIU-FORUM) Gaithersburg, MD August 6–9, 1990

**DOI:** 10.6028/jres.095.056

**Published:** 1990

**Authors:** Elizabeth B. Lennon

**Affiliations:** National Computer Systems Laboratory, National Institute of Standards and Technology, Gaithersburg, MD 20899

The National Computer Systems Laboratory (NCSL), National Institute of Standards and Technology, sponsored and hosted the North American ISDN Users’ Forum at its Gaithersburg, MD site on August 6–9, 1990. Held three times a year at various locations throughout the nation, the NIU-Forum traditionally attracts about 300 vendors and users of ISDN. NCSL and industry established the NIU-Forum in 1988 to create a strong user voice in the implementation of ISDN applications and to ensure that emerging ISDN technology meets users’ applications needs. Although the forum focuses on the requirements of ISDN users in North America, membership is open to all interested users, product providers, and service providers.

## 1. What is ISDN?

The Integrated Services Digital Network (ISDN) is a group of international standards for a worldwide communications network for the exchange of voice, data, and image information among all users, independent of any manufacturer, service provider, or implementation technology. ISDN standards are developed by the International Telephone and Telegraph Consultative Committee (CCITT) and, for North America in particular, by the Exchange Carriers Standards Association’ (ECSA) accredited standards committee, Tl, under the umbrella of the American National Standards Institute (ANSI).

The result is a set of standards with a tremendous variety of options and parameters to meet all possible needs and technologies for which the standards could be used. To ensure interoperability and terminal portability within the ISDN network and its attendant equipment, a uniform subset of options and parameters must be selected for implementation. Each application usually requires only a subset of total functionality available in the standards; for ISDN products and services to work together in a multi-vendor environment, common sets of options must be selected.

To cope with this proliferation of choices and to provide practical products and services which meet users’ needs, the standards specification process must be extended to include the development of Application Profiles, Implementation Agreements, and Conformance Criteria which will promote interoperability. The NIU-Forum addresses all of these areas.

## 2. NIU-Forum Objectives

The NIU-Forum seeks to achieve three principal objectives:

- To promote an ISDN forum committed to providing users the opportunity to influence developing ISDN technology to reflect their needs;
- To identify ISDN applications, develop implementation requirements, and facilitate their timely, harmonized, and interoperable introduction; and
- To solicit user, product provider, and service provider participation in the process.

## 3. NIU-Forum Organization

The actual work of the NIU-Forum is accomplished in two workshops: the ISDN User’s Workshop (IUW) and the ISDN Implementor’s Workshop (IIW). The IUW produces Application Requirements which describe potential applications of ISDN and the features which may be needed. The IIW develops Application Profiles, Implementation Agreements, and Conformance Criteria which provide the detailed technical decisions necessary to implement an application requirement in an interoperable manner. The NIU-Forum Executive Steering Committee coordinates the activities within the two workshops.

## 4. Accomplishments of August 1990 NIU-Forum

Specific accomplishments from the August 1990 NIU-Forum include the following:

- The IUW logged in 10 new applications for development of Application Profiles by the IIW; one previous application was revised. This action brings the total number of applications submitted in the NIU-Forum to 110;
- The IUW elected to add a new family, Security, to the applications family groups;
- Four Applications Profiles for Call Management were submitted to become stable implementation agreements;
- Stable agreements will be published as a NIST Special Publication after the November 1990 meeting of the NIU-Forum; and
- The NIU-Forum plans a nationwide, possibly worldwide, demonstration of ISDN technology for October 1991. The demonstration will highlight ISDN products and services implementing specifications agreed to in forum workshops.

